# A cross-sectional study of the association between a novel integrated insulin resistance indicator (IR6) and anxiety: Data from NHANES 2007 to 2012

**DOI:** 10.1097/MD.0000000000048848

**Published:** 2026-05-15

**Authors:** Ya He, Ya Lan, Xuelian Li, Fei Wang, Jingang Li, Hui Zhou, Yuetao Wen

**Affiliations:** a Department of Physical Examination Center, Chongqing University Jiangjin Hospital, Chongqing, China; b Department of Neurosurgery, Chongqing University Jiangjin Hospital, Chongqing, China.

**Keywords:** anxiety, body mass index (BMI), homeostasis model assessment of β cell function (HOMA-β), insulin resistance, NHANES, triglyceride-glucose (TyG)

## Abstract

The increasing association of insulin resistance (IR) with severe mental illnesses has a lot of importance to mental health and metabolism. Researching the association between IR indices and anxiety is quite fragmented. This study included 1670 individuals with anxiety and 4994 individuals without anxiety from 2007 to 2012 National Health and Nutrition Examination Survey Database. A novel IR indicator (IR6) was developed by combining 6 routine IR-related clinical indices (age, body mass index [BMI], insulin, fasting blood glucose, triglycerides, and total cholesterol). Afterward, we used multivariate logistic regression models, correlation analysis, restricted cubic spline, and subgroup analysis to systematically evaluate the relationship between IR6 and anxiety. The findings indicated that IR6 levels were significantly higher in anxious participants than in non-anxious participants. This pattern was the same as that of triglyceride-glucose (TyG), TyG–BMI, homeostatic model assessment of IR (HOMA-IR), and HOMA-β. The study found a strong positive link between IR6 and TyG, TyG–BMI, HOMA-IR, and HOMA-β. According to the findings of multivariable logistic regression analyses, increased IR6 levels were an independent risk for anxiety, and this relationship remained statistically significant in the subgroup analyses according to the other variables. Restricted cubic spline analysis also found that IR6 had a positive nonlinear relationship with anxiety. In conclusion, our study confirms a strong relationship between IR and anxiety. Moreover, we reveal that IR6 is a novel IR marker with promise as a tool for anxiety risk assessment. Our knowledge of the relationship between metabolic function and mental health is further investigated with these findings.

## 1. Introduction

Global prevalence of anxiety disorder is rated one of the highest among mental disorders. Anxiety disorders usually tend to onset in early adulthood or even earlier. The global incidence of anxiety has shown a steady increase rate in recent years.^[1]^ The World Health Organization states that 3 billion people globally suffer from anxiety symptoms, which equals 3.3% of the global burden of disease. This further indicates that anxiety disorders can have a debilitating effect on an individual and health systems in society.^[1,2]^

Anxiety disorders are associated with several physical health problems. Moreover, they have the capacity to produce severe distress but can also interfere with social and occupational functioning. Clinical evidence and epidemiological studies consistently show that people with diabetes, obesity, or metabolic syndrome have a higher prevalence of anxiety disorders compared to the general population. Likewise, anxiety symptoms are also highly prevalent among patients with these metabolic diseases.^[3–10]^ This bidirectional relationship is characterized as comorbidity, which refers to a greater than expected incidence of 1 disease in those who have another disease or disorder. The existence of comorbidity not only ups the disease burden but also leads to a lesser treatment response with a poor prognosis for the affected individual and thus there is an urgent need for targeted clinical intervention strategies.^[11]^ Thus, it is important to know how these metabolic conditions may predispose to anxiety for better outcomes. Crucially, this underscores the need to identify metabolic markers that link the assessments of body and mind. Insulin resistance (IR) is a pivotal characteristic of metabolic syndrome among these potential metabolic markers. This makes it a particularly pertinent candidate for the investigation of metabolic disturbances and anxiety.

IR is the impaired ability of insulin to regulate glucose metabolism in target tissues (e.g., skeletal muscle, adipose tissue, and liver). These are common metabolic derangements that normally occur together with each other, such as hyperinsulinemia, hyperglycemia, hyperlipidemia, and obesity.^[12]^ According to new studies, IR and anxiety could be related to each other. As insulin sensitivity improves, anxiety does decrease significantly in the individual. This indicates that IR may regulate anxiety development and progression.^[13]^ Previous research has shown that indicators related to IR are associated with anxiety, including basic metabolic parameters such as fasting blood glucose (FBG), body mass index (BMI), insulin, triglycerides (TG), high-density lipoprotein cholesterol (HDL-C), and low-density lipoprotein cholesterol (LDL-C), as well as commonly used IR evaluation indices such as the TG–BMI, and homeostatic model assessment of IR (HOMA-IR).^[14–17]^

However, current approaches to assessing IR and exploring its connection to anxiety are quite limited. First, existing indicators for IR is based on either 1 or a few metabolic parameters. For instance; the HOMA-IR depends on FBG and insulin. Similarly, the triglyceride-glucose (TyG) also depends on FBG and TG. The overlap of parameters or the missing of key dimensions creates redundancy and incomprehensiveness when using multiple indicators together resulting in distortions in analyses and masking the true relationship between IR and anxiety. Second, current research lacks integrated IR indicators. The use of a single indicator in most studies to analyze the association of IR with anxiety does not take into account the various factors that together reflect IR status simultaneously. This limits the ability of the researchenter to capture the complexity of IR and clarify the overall impact of IR on anxiety. Moreover, the result credibility and generalizability is further undermined by the limited number of studies assessing the dose–response effect of IR on anxiety.

To address these gaps, we conducted a cross-sectional study using data from the National Health and Nutrition Examination Survey (NHANES) to assess the relationship between IR and anxiety. We also developed a novel exploratory integrated IR-related metabolic indicator by combining routine clinical indices and examined its cross-sectional association with anxiety in this study population. The purpose of this study is to enhance the validity of anxiety risk assessment tools, provide fresh insights into the metabolic mechanism of anxiety, advance the transformation of clinical anxiety’s diagnostic approach from “single indicator detection” to “integrated indicator precise assessment,” and at the same time, provide a reference paradigm for the development of integrated indicators for the follow-up metabolic-related mental disorders in the future.

## 2. Materials and methods

### 2.1. Study population

**Figure 1.**
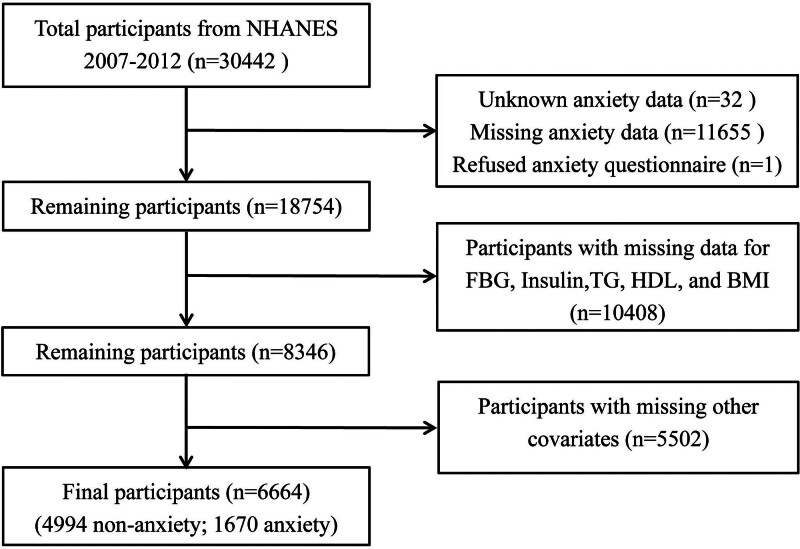
Study population screening flow chart.

NHANES employs a multi-phase sampling approach to guarantee demographic diversity within cohorts, subsequently gathering data through a standardized questionnaire for physical examinations and dietary assessments – facilitating a comprehensive understanding of the health and nutrition of the U.S. population.^[18]^ In this study, we gathered epidemiological information from the NHANES survey cycles conducted between 2007 and 2012 via the official website (https://www.cdc.gov/nchs/nhanes/). Participants were excluded based on the following criteria: missing or unknown data on anxiety (n = 11,688); missing data on FBG, TG, insulin, HDL-C, or BMI (n = 10,488); missing information on other covariates (n = 5502); and age <20 years. Finally, 6664 eligible participants were included in the analysis (Fig. 1).

### 2.2. Definition of anxiety

In this study, anxiety status was assessed by the following question, “during the past 30 days, for about how many days have you felt worried, tense, or anxious?” Individuals reporting anxiety on 0 to 6 days per month were assigned to the non-anxious group, whereas those experiencing anxiety on 7 to 30 days per month were designated as the anxious group.^[19,20]^

### 2.3. Covariables

Covariate data were extracted from 3 NHANES datasets: age, gender, race (Mexican American, non-Hispanic Black, non-Hispanic White, other Hispanic, and other race-including multi-racial), and education level (9–11th grade, college graduate or above, high school graduate, <9th grade, and some college or AA degree) were retrieved from the demographics dataset; alcohol consumption status (individuals consuming at least 12 alcoholic drinks annually were defined as alcohol users), smoking status (individuals who had smoked at least 100 cigarettes in their lifetime were defined as smokers), hypertension, and diabetes were obtained from the questionnaire dataset; and BMI (kg/m^2^), glycated hemoglobin (HbA1C, %), FBG (mg/dL and mmol/L), fasting insulin (μU/mL), HDL-C (mg/dL), LDL-C (mg/dL), total cholesterol (TC, mg/dL), and TG (mg/dL) were derived from the laboratory dataset.

### 2.4. Definition of IR indices

The quantitative insulin sensitivity check index (QUICKI) was calculated as follows: QUICKI = 1/[log(fasting insulin, μU/mL) + log(FBG, mg/dL)]. The HOMA-IR was calculated as follows: HOMA-IR = FBG (mmol/L) × fasting insulin (μU/mL)/22.5. The homeostasis model assessment of β-cell function (HOMA-β) was calculated as follows: HOMA-β = 20 × fasting insulin (μU/mL)/[FBG (mmol/ml) − 3.5]. The TyG index was calculated as follows: TyG = Ln [TG (mg/dL) × FBG (mg/dL)/2]. The TyG–BMI index was calculated as follows: TyG–BMI = TyG index × BMI (kg/m^2^).^[21]^

### 2.5. Definition of novel IR indicator

Our findings suggest that anxiety is associated with a range of parameters, which include age, BMI, FBG, insulin, TC, TG, QUICKI, HOMA-IR, HOMA-β, TyG, and TyG–BMI. Notably, QUICKI, HOMA-IR, HOMA-β, TyG, and TyG–BMI are derived from the core baseline indices BMI, FBG, insulin, and TG. To overcome current limitations, a new IR indicator called IR6 (insulin resistance 6-factor index) has been developed based on 6 routine clinical factors (age, BMI, insulin, FBG, TG, and TC). To calculate IR6, the following formula is followed: IR6 = log_10_ [BMI (kg/m^2^) × FBG (mg/dL) × insulin (μU/mL) × TG (mg/dL) × TC (mg/dL)/age (years)]. This equation is based on a ratio. The numerator is made up of the product of 5 variables, namely, BMI, FBG, insulin, TG, and TC. These were seen to have a positive correlation with the anxiety status. The denominator is age, which had a negative correlation with the anxiety status.

### 2.6. Statistical analysis

This study utilized R software (version 4.5.0; R Foundation for Statistical Computing, Vienna, Austria) and GraphPad Prism (version 10.4.2; GraphPad Prism Software Inc., San Diego) for statistical analysis, incorporating the complex survey design and weighting procedures of NHANES data. The basic features of the research participants were classified independently, either based on anxiety status or according to tertile categories of IR6. Continuous variables are represented by their mean and standard deviation (SD), while categorical variables are shown as frequency alongside their respective percentage. The Kruskal–Wallis test was employed for comparing continuous variables, whereas the Pearson chi-square test was utilized for assessing categorical variables. To enhance the precision of the data and minimize the impact of the intricate multi-stage sampling design employed in NHANES, the research applied sample weights as recommended by the NHANES guidelines. All statistical analyses accounted for the complex sampling design of NHANES, with WTMEC2YR used as the core weight for all analyses including descriptive statistics, correlation analysis, logistic regression models, restricted cubic spline (RCS) analysis, and subgroup analysis. Stratification (SDMVSTRA) and primary sampling unit (SDMVPSU) were also integrated to adjust for sampling bias, non-response error, and the oversampling of specific populations, and the WTMEC2YR was standardized by cycle for the pooled analysis of the 2007 to 2012 NHANES data.

We built Cox proportional hazards regression models at 3 levels of adjustment for confounding factors to assess the independent predictive value of IR6. Unadjusted model 1; model 2 adjusted for gender, age, race, education level, alcohol consumption status and smoking status; model 3 further incorporated HbA1C, HDL-C, and LDL-C based on model 2. Generalized variance inflation factor (GVIF) analysis was conducted to detect multicollinearity among covariates in the regression models. Covariates with GVIF values above 10 were considered to exhibit significant collinearity.

To assess potential trends for associations, analyses were also performed using the continuous IR6 variable as tertile categories, with tertile 1 (T1) chosen as the reference group. RCS curves were formed to evaluate the dose–response association of the IR6 and anxiety status. The analysis of subgroups based on relevant variables was also analyzed for their association with IR6 and anxiety discussing interaction effects. A *P* value <.05 was statistically significant.

## 3. Results

### 3.1. Baseline characteristics of participants categorized by anxiety status

The current study involved 1670 participants with anxiety diagnosis and 4994 non-anxious participants in total. Table 1 compares the baseline features of individuals with anxiety to those without anxiety. Several variables were significantly associated with anxiety status (*P* < .05), including demographic factors (gender, age, race, and education level), lifestyle-related indicators (smoking status), clinical conditions (hypertension and diabetes status), as well as, anthropometric and metabolic parameters (BMI, FBG, insulin, TC, TG, TyG, TyG–BMI, HOMA-IR, and HOMA-β). We find that, compared with non-anxious participants, anxious participants are younger and have higher BMI, FBG, insulin, TC, TG, TyG, TyG–BMI, HOMA-IR, and HOMA-β.

**Table 1:** Baseline characteristics of participants categorized by anxiety status.

		Overall (n = 6664)	Non-anxiety (n = 4994)	Anxiety (n = 1670)	*P*
Gender (%)	Female	3376 (50.7)	2340 (46.9)	1036 (62.0)	<.001
	Male	3288 (49.3)	2654 (53.1)	634 (38.0)	
Age (yr)		49.88 (17.81)	50.54 (18.18)	47.92 (16.50)	<.001
Race (%)	Mexican American	1052 (15.8)	804 (16.1)	248 (14.9)	.004
	Non-Hispanic Black	1287 (19.3)	972 (19.5)	315 (18.9)	
	Non-Hispanic White	3073 (46.1)	2274 (45.5)	799 (47.8)	
	Other Hispanic	738 (11.1)	530 (10.6)	208 (12.5)	
	Other race – including multi-racial	514 (7.7)	414 (8.3)	100 (6.0)	
Education (%)	9–11th grade	1048 (15.7)	734 (14.7)	314 (18.8)	<.001
	College graduate or above	1502 (22.5)	1179 (23.6)	323 (19.3)	
	High school graduate	1521 (22.8)	1153 (23.1)	368 (22.0)	
	Less than 9th grade	763 (11.4)	580 (11.6)	183 (11.0)	
	Some college or AA degree	1830 (27.5)	1348 (27.0)	482 (28.9)	
Alcohol (%)	No	1823 (27.4)	1373 (27.5)	450 (26.9)	.687
	Yes	4841 (72.6)	3621 (72.5)	1220 (73.1)	
Smoke (%)	No	3629 (54.5)	2793 (55.9)	836 (50.1)	<.001
	Yes	3035 (45.5)	2201 (44.1)	834 (49.9)	
Hypertension (%)	No	4240 (63.6)	3239 (64.9)	1001 (59.9)	<.001
	Yes	2424 (36.4)	1755 (35.1)	669 (40.1)	
Diabetes (%)	No	5722 (85.9)	4333 (86.8)	1389 (83.2)	
	Borderline	132 (2.0)	89 (1.8)	43 (2.6)	
	Yes	810 (12.2)	572 (11.5)	238 (14.3)	
BMI (kg/m^2^)		28.86 (6.62)	28.66 (6.34)	29.44 (7.38)	<.001
HbA1C (%)		5.74 (1.03)	5.73 (0.99)	5.78 (1.14)	.142
FBG (mg/dL)		107.99 (32.97)	107.27 (30.82)	110.14 (38.63)	.002
FBG (mmol/L)		5.99 (1.83)	5.95 (1.71)	6.11 (2.14)	.002
Insulin (μU/mL)		13.78 (15.03)	13.33 (12.47)	15.15 (20.83)	<.001
HDL-C (mg/dL)		53.91 (15.65)	53.86 (15.43)	54.05 (16.27)	.677
LDL-C (mg/dL)		115.36 (35.35)	115.09 (35.07)	116.17 (36.18)	.282
Total cholesterol (mg/dL)		193.99 (40.57)	193.36 (40.17)	195.88 (41.70)	.028
TG (mg/dL)		123.59 (65.57)	122.00 (64.26)	128.33 (69.14)	.001
TyG		8.65 (0.60)	8.63 (0.59)	8.69 (0.63)	<.001
TyG–BMI		250.68 (64.63)	248.44 (61.46)	257.37 (72.90)	<.001
HOMA-IR		3.87 (5.43)	3.70 (4.58)	4.41 (7.39)	<.001
QUICKI		0.40 (0.02)	0.40 (0.02)	0.40 (0.02)	.167
HOMA-β		42.70 (43.97)	41.50 (36.97)	46.27 (60.10)	<.001

BMI = body mass index, FBG = fasting blood glucose, HbA1C = glycated hemoglobin, HDL-C = high-density lipoprotein cholesterol, HOMA-IR = homeostatic model assessment of insulin resistance, HOMA-β = homeostasis model assessment of β-cell function, IR = insulin resistance, IR6 = insulin resistance 6-factor index, LDL-C = low-density lipoprotein cholesterol, QUICKI = quantitative insulin sensitivity check index, RCS = restricted cubic spline, TC = total cholesterol, TG = triglycerides, TyG = triglyceride-glucose, TyG–BMI = triglyceride-glucose–body mass index.

### 3.2. Characteristics of participants stratified by IR6 tertiles

Statistical analyses revealed that age, BMI, FBG, insulin, TC, TG, TyG, TyG–BMI, HOMA-IR, and HOMA-β were factors associated with anxiety status. TyG, TyG–BMI, HOMA-IR, and HOMA-β are mainly derived from routine measurable indicators (i.e., BMI, FBG, insulin, and TG). Current indicators related IR such as TyG, HOMA-IR, etc share some overlapping information. For instance, both share FBG and insulin, which can lead to redundant information. This redundancy may, in turn, affect the accuracy of further analysis. In order to alleviate this overlap and develop a better integrated metric, we proposed a novel IR indicator namely IR6, calculated using the following equation: IR6 = log_10_ [BMI (kg/m^2^) × FBG (mg/dL) × insulin (μU/mL) × TG (mg/dL) × TC (mg/dL)/age (years)]. Participants with anxiety exhibited a significantly higher IR6 level than those without anxiety, with values of 7.21 ± 0.58 and 7.12 ± 0.54, respectively (*P* < .001). The IR6 was further classified into 3 tertiles T1 5.197–6.880, T2 6.880–7.368, and T3 7.368–9.386. The overall characteristics of participants stratified by IR6 tertiles (Table 2). The prevalence of anxiety increased with increasing IR6 levels which was significantly different with 22.7% in T1 (lowest IR6) group and 28.1% in T3 (highest IR6) group (*P* < .001, Table 2). In addition, participants with greater IR6 levels, were more likely to be male and younger and had greater hypertension and diabetes prevalence (Table 2). The patients with higher IR6 showed other significantly higher in BMI, HbA1C, FBG, insulin, HDL, LDL, TC, TG, TyG, TyG–BMI, HOMA-IR, QUICKI, and HOMA-β (Table 2).

**Table 2:** Characteristics of participants stratified by IR6 tertiles.

	Level	Overall (n = 6664)	T1 (n = 2222) (5.197–6.880)	T2 (n = 2221) (6.880–7.368)	T3 (n = 2221) (7.368–9.386)	*P*
Anxiety (%)	No	4994 (74.9)	1717 (77.3)	1681 (75.7)	1596 (71.9)	<.001
	Yes	1670 (25.1)	505 (22.7)	540 (24.3)	625 (28.1)	
Gender (%)	Female	3376 (50.7)	1172 (52.7)	1141 (51.4)	1063 (47.9)	.004
	Male	3288 (49.3)	1050 (47.3)	1080 (48.6)	1158 (52.1)	
Age (yr)		49.88 (17.81)	52.47 (18.53)	50.70 (17.97)	46.48 (16.32)	<.001
Race (%)	Mexican American	1052 (15.8)	222 (10.0)	339 (15.3)	491 (22.1)	<.001
	Non-Hispanic Black	1287 (19.3)	460 (20.7)	444 (20.0)	383 (17.2)	
	Non-Hispanic White	3073 (46.1)	1146 (51.6)	1005 (45.2)	922 (41.5)	
	Other Hispanic	738 (11.1)	194 (8.7)	264 (11.9)	280 (12.6)	
	Other Race – including multi-racial	514 (7.7)	200 (9.0)	169 (7.6)	145 (6.5)	
Education (%)	9–11th grade	1048 (15.7)	325 (14.6)	317 (14.3)	406 (18.3)	<.001
	College graduate or above	1502 (22.5)	639 (28.8)	507 (22.8)	356 (16.0)	
	High school graduate	1521 (22.8)	469 (21.1)	521 (23.5)	531 (23.9)	
	Less than 9th grade	763 (11.4)	211 (9.5)	261 (11.8)	291 (13.1)	
	Some college or AA degree	1830 (27.5)	578 (26.0)	615 (27.7)	637 (28.7)	
Alcohol (%)	No	1823 (27.4)	595 (26.8)	618 (27.8)	610 (27.5)	.729
	Yes	4841 (72.6)	1627 (73.2)	1603 (72.2)	1611 (72.5)	
Smoke (%)	No	3629 (54.5)	1218 (54.8)	1229 (55.3)	1182 (53.2)	.337
	Yes	3035 (45.5)	1004 (45.2)	992 (44.7)	1039 (46.8)	
Hypertension (%)	No	4240 (63.6)	1542 (69.4)	1398 (62.9)	1300 (58.5)	<.001
	Yes	2424 (36.4)	680 (30.6)	823 (37.1)	921 (41.5)	
Diabetes (%)	Borderline	132 (2.0)	30 (1.4)	48 (2.2)	54 (2.4)	<.001
	No	5722 (85.9)	2005 (90.2)	1908 (85.9)	1809 (81.4)	
	Yes	810 (12.2)	187 (8.4)	265 (11.9)	358 (16.1)	
BMI (kg/m^2^)		28.86 (6.62)	24.72 (4.32)	28.40 (4.99)	33.46 (7.03)	<.001
HbA1C (%)		5.74 (1.03)	5.55 (0.71)	5.67 (0.87)	6.01 (1.33)	<.001
FBG (mg/dL)		107.99 (32.97)	98.91 (20.81)	105.75 (25.67)	119.30 (44.21)	<.001
FBG (mmol/L)		5.99 (1.83)	5.49 (1.16)	5.87 (1.42)	6.62 (2.45)	<.001
Insulin (μU/mL)		13.78 (15.03)	6.13 (2.88)	11.47 (5.25)	23.76 (21.88)	<.001
HDL-C (mg/dL)		53.91 (15.65)	61.85 (16.61)	53.91 (13.97)	45.96 (11.72)	<.001
LDL-C (mg/dL)		115.36 (35.35)	104.34 (31.35)	116.82 (34.34)	124.93 (37.06)	<.001
Total cholesterol (mg/dL)		193.99 (40.57)	181.97 (37.90)	194.01 (38.97)	206.00 (41.18)	<.001
TG (mg/dL)		123.59 (65.57)	78.93 (31.12)	116.34 (46.10)	175.51 (71.16)	<.001
TyG		8.65 (0.60)	8.18 (0.43)	8.63 (0.43)	9.13 (0.51)	<.001
TyG–BMI		250.68 (64.63)	202.23 (36.90)	244.81 (42.56)	305.01 (63.45)	<.001
HOMA-IR		3.87 (5.43)	1.50 (0.77)	3.00 (1.54)	7.13 (8.28)	<.001
QUICKI		0.40 (0.02)	0.40 (0.02)	0.40 (0.02)	0.41 (0.02)	<.001
HOMA-β		42.70 (43.97)	19.32 (11.10)	36.83 (20.38)	71.95 (61.86)	<.001

BMI = body mass index, FBG = fasting blood glucose, HbA1C = glycated hemoglobin, HDL-C = high-density lipoprotein cholesterol, HOMA-IR = homeostatic model assessment of insulin resistance, HOMA-β = homeostasis model assessment of β-cell function, IR = insulin resistance, IR6 = insulin resistance 6-factor index, LDL-C = low-density lipoprotein cholesterol, QUICKI = quantitative insulin sensitivity check index, RCS = restricted cubic spline, TC = total cholesterol, TG = triglycerides, TyG = triglyceride-glucose, TyG–BMI = triglyceride-glucose–body mass index.

### 3.3. The correlation between IR6 and variables

**Figure 2.**
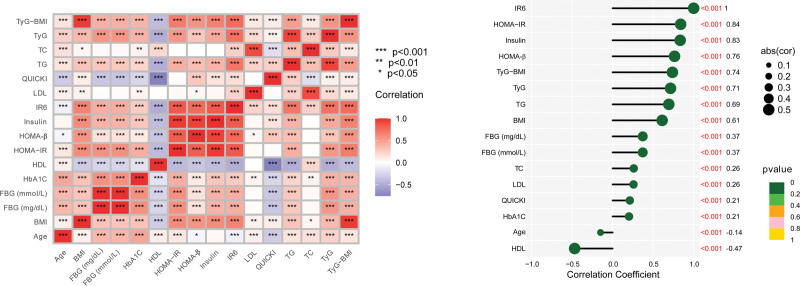
The correlation between IR6 and variables. IR6 = insulin resistance 6-factor index.

The relationship between IR6 was investigated and correlation analysis done whose outcome has been shown in Figure 2. IR6 exhibited a strong positive correlation with HOMA-IR, insulin, HOMA-β, TyG–BMI, and TyG. The discovery conclude that IR6 is a universal indicator which can precisely demonstrate IR status. On the contrary, IR6 was negatively correlated with age and HDL. However, the strength of the correlations was weak.

### 3.4. Associations between IR6 and anxiety

**Figure 3.**
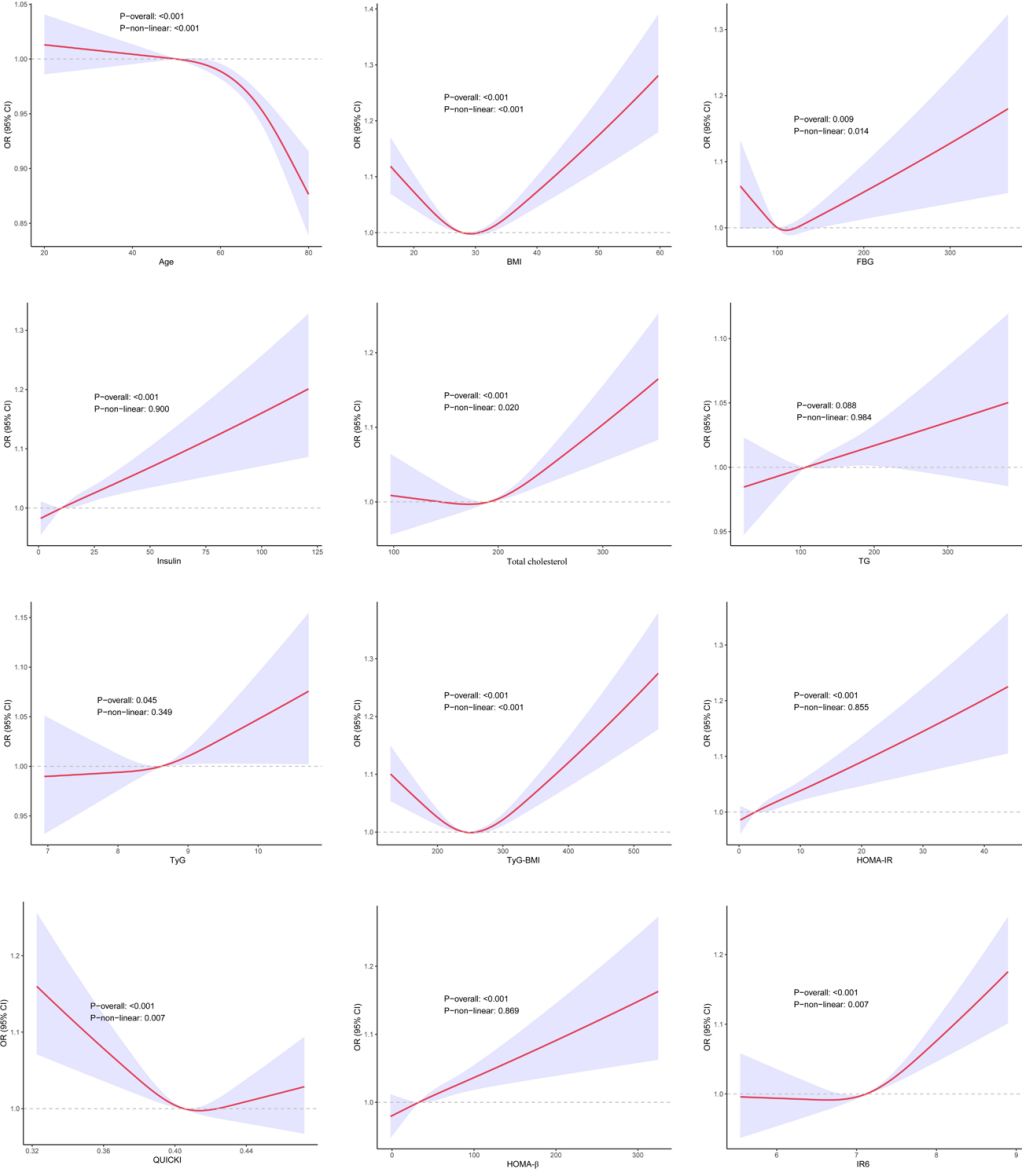
Weighted RCS curves for association between IR6 and variables. IR6 = insulin resistance 6-factor index; RCS = restricted cubic spline.

**Figure 4.**
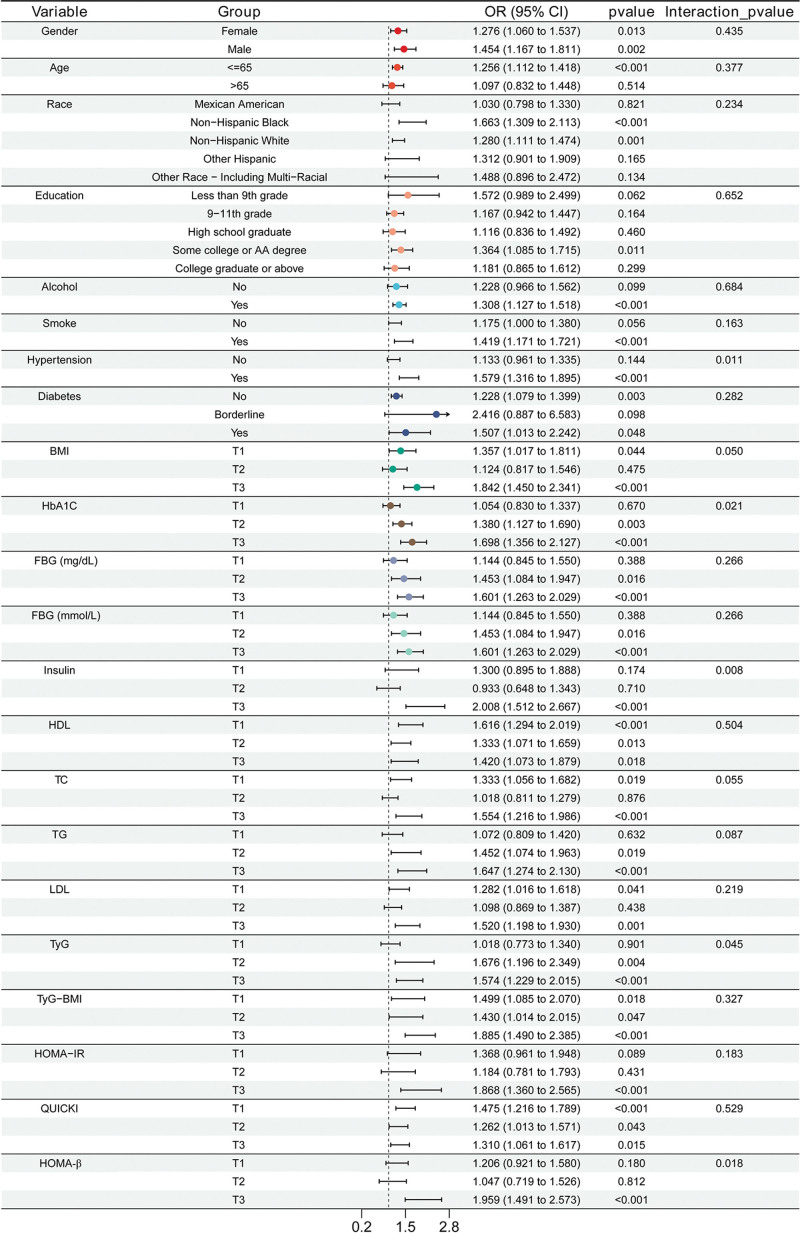
Subgroup analysis of the association between IR6 and the prevalence of anxiety. CI = 95% confidence intervals; IR6 = insulin resistance 6-factor index; OR = odds ratio.

To address potential multicollinearity, GVIF diagnostics were conducted for all variables in model 2 (Table S1, Supplemental Digital Content) and model 3 (Table S2, Supplemental Digital Content), and all GVIF values were <10, confirming no severe multicollinearity. We used logistic regression to examine the association between IR6 and anxiety. The results are presented in Table 3. When using IR6 as a continuous predictor, the fully adjusted model 3 revealed an increased likelihood of anxiety by 32% (OR 1.32; 95% CI: 1.13–1.54; *P* < .05) with each unit increase in IR6. It is remarkable that this positive association remained statistically significant in all other adjusted models – an OR of 1.29 (95% CI: 1.15–1.45) in model 1 and an OR of 1.27 (95% CI: 1.11–1.44) in model 2. Moreover, in the analysis of IR6 as a categorical variable (tertiles), the highest IR6 tertile (T3) had a consistently higher prevalence of anxiety across all models than the lowest (T1). Together, these findings indicate that increased IR6 is an independent risk for anxiety. This finding is further confirmed by RCS analysis, which showed an upward nonlinear relationship of IR6 with anxiety (Fig. 3). The analysis conducted using RCS revealed that many other indicators related to IR (TyG, TyG–BMI, HOMA-IR, and HOMA-β) were also associated with anxiety. However, the association forms were not dissimilar. Figure 3 details these distinct association patterns for each indicator. This observation indicates that IR will lead to anxiety. In the end, we conducted a subgroup analysis of IR6 for their respective associations with anxiety (Fig. 4). Most stratified populations showed the same pattern for the association between IR6 and anxiety, with no evidence of any statistically significant interaction (*P* for interaction >.05). The consistency of the findings indicates that subgroup characteristics do not significantly change the relationship between IR6 and anxiety. This provides further support for the validity of IR6 as an independent risk factor for anxiety.

**Table 3:** Associations between the IR6 and anxiety.

IR6	Model 1		Model 2		Model 3	
	OR (95% CI)	*P* value	OR (95% CI)	*P* value	OR (95% CI)	*P* value
Continuous	1.29 (1.15–1.45)	<.001	1.27 (1.11–1.44)	.001	1.32 (1.13–1.54)	.001
Tertiles						
Tertile 1	Reference		Reference		Reference	
Tertile 2	1.14 (0.98–1.33)	.106	1.14 (0.97–1.33)	.116	1.15 (0.98–1.35)	.102
Tertile 3	1.26 (1.07–1.48)	.009	1.24 (1.03–1.48)	.028	1.25 (1.01–1.53)	.046
*P* for trend	1.12 (1.03–1.22)	.009	1.11 (1.02–1.22)	.028	1.12 (1.01–1.24)	.045

Model 1 adjusts for: none.

Model 2 adjusts for: gender, age, race, education, alcohol status, and smoke status.

Model 3 adjusts for: model 2 + HbA1C, HDL-C, LDL-C.

HbA1C = glycated haemoglobin, HDL-C = high-density lipoprotein cholesterol, IR = insulin resistance, IR6 = insulin resistance 6-factor index, LDL-C = low-density lipoprotein cholesterol.

## 4. Discussion

Using a large, representative sample from the NHANES database, we found anxious individuals had significantly higher levels of most IR-related indicators (BMI, FBG, insulin, TC, TG, TyG, TyG–BMI, HOMA-IR, HOMA-β) and younger age. These results reveal a statistical association between IR-related metabolic abnormalities and anxiety in this large representative population-based sample. Consequently, we constructed a new IR indicator (IR6) from the aggregation of 6 routine IR-related clinical indices: age, BMI, insulin, FBG, TG, and TC. IR6 is strongly correlated with traditional IR indicators, confirming its validity for evaluating IR-related metabolic profiles in this study population. Notably, IR6 showed a positive association with anxiety even after adjusting for established confounders (gender, age, race, education, alcohol use, smoking status, HbA1C, HDL-C, LDL-C), and this association remained stable after multivariable adjustment. A positive dose–response cross-sectional relationship was also observed between IR6 and anxiety. In the end, subgroup analyses indicated no significant interactions, confirming the robustness of the observed cross-sectional association between IR6 and anxiety.

There has been more and more evidence to support a strong association between mental ailments and poor metabolic health in recent years indicating a strong association between metabolic comorbidity and mental disorders.^[22]^ Type 2 diabetes mellitus is one of the most common chronic metabolic conditions. There is a high rate of comorbidity with a range of mental disorders. Notably, IR (the main pathological hallmark of type 2 diabetes mellitus) has been shown to induce anxiety-like behavior in clinical and preclinical studies.^[23,24]^ Consistent with this established framework, our findings show a significant cross-sectional association between diabetes mellitus and a higher prevalence of anxiety in this study population, which further supports the documented bidirectional cross-sectional relationship between metabolic status and mental health outcomes.

TyG, TyG–BMI, QUICKI, HOMA-IR, and HOMA-β are several composite indices that have been recently proposed in clinical practice as surrogate measures of IR. Values of these indicators are easily estimated from clinical parameters like FBG, TG, BMI, insulin, etc. Consistent with prior investigations, our data showed that participants with anxiety exhibited significantly elevated levels of BMI, FBG, fasting insulin, TG, TyG, TyG–BMI, and HOMA-IR compared with non-anxious participants.^[17,25,26]^ Importantly, our study also observed a significant increase in HOMA-β levels in anxious individuals compared to non-anxious individuals, a finding not previously observed in the literature. On the contrary, there was no significant difference in the QUICKI, another indicator of IR, between the 2 groups. Lipid indicators are also an essential marker for IR; therefore, besides TG, we have included TC, HDL-C, and LDL-C in the study. The increase of TC in anxious participants compared to non-anxious participants in our study was consistent with other literature. However, HDL-C and LDL-C were not significantly different between the 2 groups. More specifically, previous studies show positive correlations between levels of TG, TC, LDL-C, and anxiety. Conversely, there is a negative correlation between anxiety and levels of HDL-C. In fact, due to increased high glucose and cholesterol, zebrafish subjected to 2% glucose and 10% cholesterol showed increased the systemic glucose, TG, TC, and LDL-C but decreased the HDL-C eventually causing an anxiety-like behavior in the experimental type 2 diabetes model.^[27]^ Furthermore, a cohort study of more than 200,000 individuals showed that the presence of hyperglycemia, high TG, and low HDL is associated with subsequent anxiety risk.^[14]^ The differences in the populations used, sample size and experimental designs could account for the discrepancy between our findings and the literature.

Traditional IR indicators each capture distinct dimensions of IR-related metabolic profiles, and inconsistent findings in prior studies may reflect differences in study populations, variable distribution, measurement error, or model adjustment strategies rather than inherent methodological inferiority of the indices themselves. Many conventional IR indicators are constructed from a limited set of metabolic parameters (e.g., QUICKI and HOMA-IR from FBG and insulin; TyG from TG and FBG), and in this study, some indices (QUICKI, HDL-C, LDL-C) showed no significant difference between anxious and non-anxious groups, while others (HOMA-IR, TyG, TyG–BMI, TC) were positively associated with anxiety. Notably, QUICKI and HOMA-IR are mathematically inverse measures of insulin sensitivity and resistance, respectively, which may explain their divergent associations with anxiety in this dataset; no single index was intended to capture the full spectrum of IR-related metabolic abnormalities associated with anxiety. Similarly, there was no association between QUICKI and HOMA-IR and QUICKI and LDL-C, which reflects that individual IR indices capture only partial aspects of IR-related metabolic variation rather than indicating instability of the indices themselves, particularly in populations with subtle metabolic perturbations such as anxious individuals. We constructed a new integrated indicator for IR which we termed IR6, based on the single indicators of anxiety identified in our study (age, BMI, insulin, FBG, TG, TC). We found that IR6 level is an independent risk for anxiety and remained statistically significant across subgroups stratified by other factors. Also, RCS analysis results indicated that there is a nonlinear positive relationship between IR6 and anxiety. Collectively, these findings suggest that IR6 may serve as a potential cross-sectional marker for identifying individuals with a higher prevalence of anxiety in this study population.

The IR6 exhibited positive correlations with a variety of indicators recognized as contributors to the progress or development of IR. These criteria included traditional indices of IR – HOMA-IR, HOMA-β, TyG, TyG–BMI, and core metabolic parameters – including BMI, FBG, insulin, TG, and TC. On the other hand, IR6 was negatively correlated with HDL-C, which usually inhibits IR. This correlation pattern aligns with the well-established roles of these indicators in regulating IR.^[28,29]^ Notably, IR6 also showed a negative correlation with age. This finding requires further elaboration as the relationship between age, IR and anxiety is complex. Research indicates that the prevalence of IR increases with advancing age.^[30–32]^ In our study, we found that older age associated lower likelihood of anxiety occurrence. This result matches existing epidemiological evidence. Studies demonstrate that the prevalence, incidence, and disease burden related to anxiety is generally at its peak between the ages of 15 and 30.^[33–35]^ After this, there is a significant decrease in anxiety levels as people age, with the most minimal levels being exhibited by those above 60 years old.^[33–35]^ This trend aligns with our mean study population age of around 49.88 years. We used age as a negative factor in the IR6 calculation to better reflect the cross-sectional association between metabolic parameters and anxiety prevalence in this population. However, we must realize that age can affect anxiety in many different ways. If follow-up studies take place in other populations such as adolescents or young adults, things may change. People in these groups have a higher prevalence of anxiety, and age may be positively associated to anxiety.

The above outcomes allow us to substantiate IR6’s rationality and applicability in the assessment of anxiety risk. By incorporating indicators that align with IR’s pathophysiological characteristics and accounting for age’s population-specific effect on anxiety, IR6 aims to reflect the complex interplay of multiple IR-related metabolic factors in a single integrated metric, with its comparative performance yet to be established. First, the fact that the sample was limited to a middle-aged population (mean age about 49.88 years) restricts the generalizability of IR6 age-weighting strategy to other age groups particularly adolescents and young adults. Second, given the cross-sectional nature of the NHANES dataset, exposure (IR6) and outcome (anxiety) were measured at the same time point, which does not allow for determination of temporal order. Thus, reverse causality cannot be ruled out, and the identified association should be interpreted as a cross-sectional relationship rather than evidence of causation. Third, the assessment of anxiety was based on a single self-reported item rather than validated diagnostic tools (e.g., GAD-7) for clinical anxiety disorders, which may lead to potential bias in the identification of anxiety status. Fourth, this study did not verify the fasting status of participants when collecting metabolic indicators, and there is inherent measurement variability in IR markers in population-based surveys, which may affect the accuracy of IR-related index calculations including IR6. Fifth, as a novel integrated IR indicator, IR6 has only been internally validated in this NHANES cohort and currently lacks external validation in other independent populations; the generalizability of IR6 thus needs to be further confirmed by subsequent studies.

## 5. Conclusion

In conclusion, IR6 is a novel exploratory integrated IR-related metabolic indicator that integrates multiple routine clinical parameters to reflect the multi-dimensional nature of IR-associated metabolic profiles. It captures the statistical interrelationships among metabolic indicators, age, and anxiety prevalence in this study population, and may serve as a preliminary exploratory metric for future research investigating the IR–anxiety association. Further validation in independent datasets and comparative performance analyses with traditional IR indices are required for subsequent research.

## Acknowledgments

We thank the NHANES database for sharing the data.

## Author contributions

**Conceptualization:** Yuetao Wen.

**Data curation:** Ya He, Ya Lan, Xuelian Li.

**Methodology:** Yuetao Wen.

**Software:** Fei Wang, Jingang Li, Hui Zhou.

**Validation:** Yuetao Wen.

**Visualization:** Yuetao Wen.

**Writing – original draft:** Ya He, Ya Lan, Xuelian Li.

**Writing – review & editing:** Yuetao Wen.





## References

[1] Penninx BW Pine DS Holmes EA Reif A. Anxiety disorders. Lancet. 2021;397:914–27. 33581801 10.1016/S0140-6736(21)00359-7 PMC9248771

[2] Leichsenring F Heim N Steinert C. A review of anxiety disorders. JAMA. 2023;329:1315–6. 10.1001/jama.2023.2428 37071102

[3] Basiri R Seidu B Rudich M. Exploring the Interrelationships between diabetes, nutrition, anxiety, and depression: implications for treatment and prevention strategies. Nutrients. 2023;15:4226. 37836510 10.3390/nu15194226 PMC10574484

[4] Galler A Tittel SR Baumeister H. Worse glycemic control, higher rates of diabetic ketoacidosis, and more hospitalizations in children, adolescents, and young adults with type 1 diabetes and anxiety disorders. Pediatr Diabetes. 2021;22:519–28. 33470512 10.1111/pedi.13177

[5] Lee MK Lee SY Sohn SY Ahn J Han K Lee JH. Type 2 diabetes and its association with psychiatric disorders in young adults in South Korea. JAMA Netw Open. 2023;6:e2319132. 37389877 10.1001/jamanetworkopen.2023.19132 PMC10314316

[6] Liu S Leone M Ludvigsson JF. Association and familial coaggregation of childhood-onset type 1 diabetes with depression, anxiety, and stress-related disorders: a population-based cohort study. Diabetes Care. 2022;45:1987–93. 35913075 10.2337/dc21-1347 PMC9472496

[7] Liu S Leone M Ludvigsson JF. Early-onset type 2 diabetes and mood, anxiety, and stress-related disorders: a genetically informative register-based cohort study. Diabetes Care. 2022;45:2950–6. 36251507 10.2337/dc22-1053 PMC9862460

[8] Luo X Zhao M Zhang Y Zhang Y. Effects of baduanjin exercise on blood glucose, depression and anxiety among patients with type II diabetes and emotional disorders: a meta-analysis. Complement Ther Clin Pract. 2023;50:101702. 36423358 10.1016/j.ctcp.2022.101702

[9] Mersha AG Tollosa DN Bagade T Eftekhari P. A bidirectional relationship between diabetes mellitus and anxiety: a systematic review and meta-analysis. J Psychosom Res. 2022;162:110991. 36081182 10.1016/j.jpsychores.2022.110991

[10] Nguyen LA Pouwer F Winterdijk P. Prevalence and course of mood and anxiety disorders, and correlates of symptom severity in adolescents with type 1 diabetes: results from diabetes LEAP. Pediatr Diabetes. 2021;22:638–48. 33331108 10.1111/pedi.13174 PMC8251968

[11] McGrath IM Montgomery GW Mortlock S. Insights from Mendelian randomization and genetic correlation analyses into the relationship between endometriosis and its comorbidities. Hum Reprod Update. 2023;29:655–74. 37159502 10.1093/humupd/dmad009 PMC10477944

[12] James DE Stöckli J Birnbaum MJ. The aetiology and molecular landscape of insulin resistance. Nat Rev Mol Cell Biol. 2021;22:751–71. 34285405 10.1038/s41580-021-00390-6

[13] del Mello Gindri I Ferrari G Pinto LPS. Evaluation of safety and effectiveness of NAD in different clinical conditions: a systematic review. Am J Physiol Endocrinol Metab. 2024;326:E417–27. 37971292 10.1152/ajpendo.00242.2023

[14] Chourpiliadis C Zeng Y Lovik A. Metabolic profile and long-term risk of depression, anxiety, and stress-related disorders. JAMA Netw Open. 2024;7:e244525. 38564219 10.1001/jamanetworkopen.2024.4525 PMC10988352

[15] Mensorio MS Cebolla A Lisón JF. Emotional eating as a mediator between anxiety and cholesterol in population with overweight and hypertension. Psychol Health Med. 2017;22:911–8. 28010121 10.1080/13548506.2016.1271134

[16] Wang Q Li Y Ren H. Metabolic characteristics, prevalence of anxiety and its influencing factors in first-episode and drug-naïve major depressive disorder patients with impaired fasting glucose. J Affect Disord. 2023;324:341–8. 36586596 10.1016/j.jad.2022.12.096

[17] Wang Y Wang H Cheng B Xia J. Associations between triglyceride glucose index-related obesity indices and anxiety: insights from the national health and nutrition examination survey 2007-2012. J Affect Disord. 2025;382:443–52. 40280441 10.1016/j.jad.2025.04.134

[18] Hou XZ Lv YF Li YS. Association between different insulin resistance surrogates and all-cause mortality in patients with coronary heart disease and hypertension: NHANES longitudinal cohort study. Cardiovasc Diabetol. 2024;23:86. 38419039 10.1186/s12933-024-02173-7 PMC10903030

[19] Bai L Wen Z Zhu Y Jama HA Sawmadal JD Chen J. Association of blood cadmium, lead, and mercury with anxiety: a cross-sectional study from NHANES 2007-2012. Front Public Health. 2024;12:1402715. 39188794 10.3389/fpubh.2024.1402715 PMC11345141

[20] Dantzer JA Keet CA. Anxiety associated with food allergy in adults and adolescents: an analysis of data from the national health and nutrition examination survey (NHANES) 2007-2010. J Allergy Clin Immunol Pract. 2020;8:1743–6.e5. 31917367 10.1016/j.jaip.2019.12.028

[21] Yao Y Wang B Geng T Chen J Chen W Li L. The association between TyG and all-cause/non-cardiovascular mortality in general patients with type 2 diabetes mellitus is modified by age: results from the cohort study of NHANES 1999–2018. Cardiovasc Diabetol. 2024;23:43. 38281973 10.1186/s12933-024-02120-6 PMC10823741

[22] Chen S Wang J Lang X Zhang XY. Clinical correlates, lipid metabolic parameters and thyroid hormones are associated with abnormal glucose metabolism in first-episode and drug-naïve major depressive disorder patients with suicide attempts: a large cross-sectional study. J Affect Disord. 2025;380:10–6. 40120949 10.1016/j.jad.2025.03.102

[23] Bui BT Florentin D Fournier F Ploux O Méjean A Marquet A. Biotin synthase mechanism: on the origin of sulphur. FEBS Lett. 1998;440:226–30. 9862460 10.1016/s0014-5793(98)01464-1

[24] Martin H Bullich S Martinat M. Insulin modulates emotional behavior through a serotonin-dependent mechanism. Mol Psychiatry. 2024;29:1610–9. 36207585 10.1038/s41380-022-01812-3

[25] Han Y Zhou Z Zhang Y Zhao G Xu B. The association of surrogates of insulin resistance with hyperuricemia among middle-aged and older individuals: a population-based nationwide cohort study. Nutrients. 2023;15:3139. 37513557 10.3390/nu15143139 PMC10385684

[26] Jaremka LM Pacanowski CR. Social anxiety symptoms moderate the link between obesity and metabolic function. Psychoneuroendocrinology. 2019;110:104425. 31542635 10.1016/j.psyneuen.2019.104425 PMC7384604

[27] Wang J Li Y Lai K. High-glucose/high-cholesterol diet in zebrafish evokes diabetic and affective pathogenesis: the role of peripheral and central inflammation, microglia and apoptosis. Prog Neuropsychopharmacol Biol Psychiatry. 2020;96:109752. 31446160 10.1016/j.pnpbp.2019.109752

[28] Oliveri A Rebernick RJ Kuppa A. Comprehensive genetic study of the insulin resistance marker TG:HDL-C in the UK Biobank. Nat Genet. 2024;56:212–21. 38200128 10.1038/s41588-023-01625-2 PMC10923176

[29] Wang X Zhao C Feng H. Associations of insomnia with insulin resistance traits: a cross-sectional and mendelian randomization study. J Clin Endocrinol Metab. 2023;108:e574–82. 36794917 10.1210/clinem/dgad089

[30] Guo X Asthana P Gurung S. Regulation of age-associated insulin resistance by MT1-MMP-mediated cleavage of insulin receptor. Nat Commun. 2022;13:3749. 35768470 10.1038/s41467-022-31563-2 PMC9242991

[31] Vieira-Lara MA Dommerholt MB Zhang W. Age-related susceptibility to insulin resistance arises from a combination of CPT1B decline and lipid overload. BMC Biol. 2021;19:154. 34330275 10.1186/s12915-021-01082-5 PMC8323306

[32] Vieira-Lara MA Reijne AC Koshian S. Age and diet modulate the insulin-sensitizing effects of exercise: a tracer-based oral glucose tolerance test. Diabetes. 2023;72:872–83. 37204269 10.2337/db22-0746

[33] Hao Y Zhao H Bai R Xu Z Feng Y Gu H. Thirty-year trends in anxiety disorders incidence across china, japan, and republic of korea: an age-period-cohort analysis based on GBD 2021. Healthcare (Basel). 2025;13:1376. 40565403 10.3390/healthcare13121376 PMC12193347

[34] Wang Z Dou Y Yang X. Global, regional, and national burden of mental disorders among adolescents and young adults, 1990-2021: a systematic analysis for the global burden of disease study 2021. Transl Psychiatry. 2025;15:397. 41073427 10.1038/s41398-025-03623-w PMC12514266

[35] Wu Y Li X Ji X. Trends in the epidemiology of anxiety disorders from 1990 to 2021: a global, regional, and national analysis with a focus on the sociodemographic index. J Affect Disord. 2025;373:166–74. 39732404 10.1016/j.jad.2024.12.086

